# Population pharmacokinetic modeling and target attainment analyses of rezafungin for the treatment of candidemia and invasive candidiasis

**DOI:** 10.1128/aac.00916-23

**Published:** 2023-11-28

**Authors:** Stefan Roepcke, Julie Passarell, Helen Walker, Shawn Flanagan

**Affiliations:** 1 Cognigen Division of Simulations Plus, Inc., Buffalo, New York, USA; 2 Mundipharma, Cambridge, United Kingdom; 3 Cidara Therapeutics, San Diego, California, USA; University of Iowa, Iowa City, Iowa, USA

**Keywords:** population pharmacokinetics, target attainment, candidemia, invasive candidiasis, rezafungin

## Abstract

Rezafungin is a chemically and metabolically stable echinocandin with a longer half-life than other echinocandins, allowing for a once-weekly intravenous infusion versus a daily infusion. Rezafungin is approved in the US for the treatment of candidemia and/or invasive candidiasis and is in development for the prevention of invasive fungal disease caused by *Candida*, *Aspergillus*, and *Pneumocystis* spp. in immunosuppressed patients. A population pharmacokinetic (PPK) model was developed using data from five Phase 1, one Phase 2, and one Phase 3 study. The model found to best describe the available data was a three-compartment PPK model with first-order elimination characterized by the parameters clearance (CL), central volume (V1), peripheral volume (V23), intercompartmental clearance 1, and intercompartmental clearance 2. The variability model included correlated interindividual variability in CL, V1, and V23 and a proportional residual variability model. The following statistically significant covariates were identified: albumin concentrations on V23; body surface area (BSA) on CL, V1, and V23; and disease state on CL and V1. Disease states were defined as patients from the Phase 2 and Phase 3 studies and hepatically impaired subjects. Covariates of BSA, disease state, or albumin, included in the final model, were not associated with clinically meaningful changes in PK, nor were any other patient factors, indicating that a common dose regimen is adequate for all adult patients. Target attainment simulations were performed to estimate the probability of achieving PK/pharmacodynamic targets across the range of minimum inhibitory concentration values for six species of *Candida*.

## INTRODUCTION

Rezafungin is a water-soluble, chemically and metabolically stable echinocandin that has an extended half-life and therefore is differentiated from the marketed drugs in this class as it is administered once weekly by intravenous (IV) infusion versus daily infusion.

Rezafungin is approved in the US for the treatment of candidemia and/or invasive candidiasis. In addition, rezafungin is being studied as a single agent for the prevention of invasive fungal disease (ReSPECT trial; CT04368559) caused by *Candida*, *Aspergillus*, and *Pneumocystis* spp. among immunosuppressed patients, including those undergoing allogeneic stem cell transplantation and those receiving cytotoxic chemotherapy for hematologic malignancies.

Pharmacometric methods are now widely used to investigate dosing regimens and therapeutic exposure to antifungal treatments (1, 2). A recent study examined the target attainment of the three marketed echinocandins to determine the likelihood of achieving clinical success and determined that while wild-type organisms may be covered with current dosing, therapeutic exposures are unlikely to be achieved for *Candida* species with increasing minimum inhibitory concentration (MIC) values (2, 3). There are also concerns regarding the ability of the marketed echinocandins to reach deep tissue infections, specifically intra-abdominal and peritoneal candidiasis (4–6).

The need for new antifungal agents is also underscored by pathogen-related trends in *Candida* species during the past 15 years. The shifting predominance of non-albicans *Candida* species and increasing resistance to azoles and echinocandins has become increasingly problematic over the past few years, especially in two species: *Candida glabrata* and *Candida auris* (7, 8).

The Centers for Disease Control and Prevention (CDC) and other international groups have recently warned that fluconazole-resistant *Candida* spp. have the potential to pose a serious threat to public health (9–11). *C. auris* emerged as a global mycological phenomenon and quickly presented a public health issue in Europe. The European Centre for Disease Prevention and Control published a paper warning of the risks of *C. auris* in December 2016 and has also reported a recent outbreak in Italy (February 2022). The United States CDC estimates that the rates of *C. auris* resistance to azoles are ~90% and to amphotericin B are ~30%, while echinocandin resistance overall is ~5% (12). However, despite these challenges facing treatment of fungal infections, no new antifungal agents have been approved for treatment of candidemia since 2007 until rezafungin in March 2023.

The analyses reported herein describe the development of a population pharmacokinetic (PK) model for rezafungin and a probability of target attainment analysis. The population PK model builds on previous analyses of Phase 1 (13) and Phase 1 and 2 studies (14) by pooling subject data across several Phase 1 studies with data obtained from patients enrolled in Phase 2 (STRIVE trial; NCT02734862) and Phase 3 (ReSTORE trial; NCT03667690) efficacy and safety studies of rezafungin for the treatment of candidemia and/or invasive candidiasis (15, 16). The use of rezafungin PK data from a larger and more diverse population has allowed for the development of a robust population PK model with quantification of the PK of rezafungin in patients infected with *Candida* spp. In addition, formal covariate analysis has facilitated the identification of patient-specific factors associated with the interindividual variability (IIV) in rezafungin PK.

Target attainment simulations were performed to estimate the probability of achieving pharmacokinetic/pharmacodynamic (PK/PD) targets across the range of MIC values for six species of *Candida* based on the Clinical and Laboratory Standards Institute (CLSI) broth microdilution methodology (17) and build upon results previously reported with the earlier population PK model developed using Phase 1 data (18). Percent probabilities of achieving the nonclinical PK/PD targets associated with net fungal stasis and 1-log10 drop in colony-forming units (CFU) reductions (if available) from baseline for *Candida albicans*, *Candida glabrata*, *Candida parapsilosis*, *Candida auris*, *Candida tropicalis*, and *Candida dubliniensis* were calculated for rezafungin using simulated free area under the concentration-time curve from time 0 to 168 h (*f*AUC_0–168h_)/MIC ratios, following a loading dose of 400 mg, across 100 data sets, each containing 1,000 virtual patients (a total of 100,000 virtual patients). The MIC susceptibility breakpoint was determined to be the highest clinically relevant MIC value with a probability of PK/PD target attainment of at least 0.9.

## RESULTS

### Data used for PK modeling

Data from single**-** and multiple**-**dose studies in healthy volunteers and patients, with doses ranging from 50 to 1,400 mg, were included in the PK analysis. All doses of rezafungin were administered via IV infusion. The PK data set consisted of a total of 2,512 rezafungin concentration records from 277 subjects, of which five records were identified as below the lower limit of quantitation postdose and five other records were identified as above 50 µg/mL, which was the upper limit of quantitation. Those records together represent less than 0.5% of the observed concentrations and were excluded from the analysis.

Body height was missing for seven patients, and body weight was missing for five patients. In addition, the laboratory parameter aspartate aminotransferase (AST) was missing for one patient. These values were imputed using the study- and sex-specific median values.

### Subject characteristics

One hundred ten healthy subjects participated in the five clinical Phase 1 studies, and 167 patients with candidemia and/or invasive candidiasis participated in the Phase 2 and Phase 3 studies (Table 1). Sixteen of the patients (9.6%) were reported to require dialysis treatment. The age of subjects in the analysis population ranged from 20 to 89 years, with a median of 53 years. The analysis population consisted of 60.6% males and 39.4% females and was primarily Caucasian (76.5%), with smaller fractions of Black/African American (9.7%), Asian (10.1%), and other or unknown self-reported races (3.6%). The median (range) of body weight was 74.7 kg (34.0 to 154.5 kg), and the median body mass index (BMI) was 26.4 kg/m^2^ (13.7 to 64.4 kg/m^2^) at baseline.

**TABLE 1:** Summary statistics of baseline subject characteristics stratified by health status and overall^*a*^

Subject characteristic	Statistic	Healthy subjects, *n* = 94	Hepatic impairment, uninfected, *n* = 16	Patients, *n* = 167	Overall, *n* = 277
Age (year)	Mean (SD)	42.4 (10.7)	57.5 (7.0)	58.8 (15.2)	53.2 (15.5)
	Median (Min, Max)	42.5 (20, 65)	58.5 (41, 68)	59.0 (24, 89)	53.0 (20, 89)
Weight (kg)	Mean (SD)	78.80 (12.14)	93.11 (17.08)	73.62 (22.04)	76.50 (19.50)
	Median (Min, Max)	78.80 (51.1, 117.6)	88.65 (72.1, 134.2)	71.00 (34.0, 154.5)	74.70 (34.0, 154.5)
BMI (kg/m^2^)	Mean (SD)	27.79 (3.22)	30.94 (3.48)	25.65 (7.28)	26.69 (6.17)
	Median (Min, Max)	28.23 (20.2, 35.3)	31.20 (24.5, 35.4)	24.40 (13.7, 64.4)	26.39 (13.7, 64.4)
BSA (m^2^)	Mean (SD)	1.91 (0.19)	2.13 (0.24)	1.86 (0.30)	1.89 (0.27)
	Median (Min, Max)	1.90 (1.5, 2.5)	2.10 (1.8, 2.7)	1.80 (1.2, 2.7)	1.90 (1.2, 2.7)
IBW (kg)	Mean (SD)	61.62 (8.99)	65.19 (8.61)	62.49 (8.26)	62.35 (8.54)
	Median (Min, Max)	61.00 (41.5, 81.3)	67.05 (52.7, 83.0)	64.00 (38.7, 79.0)	62.90 (38.7, 83.0)
CrCL (mL/min)	Mean (SD)	120.70 (22.90)	132.73 (38.35)	99.71 (101.75)	108.74 (81.33)
	Median (Min, Max)	121.80 (66.8, 176.0)	129.20 (89.2, 245.5)	82.10 (9.3, 1097.9)	104.90 (9.3, 1097.9)
SCR (mg/dL)	Mean (SD)	0.84 (0.17)	0.79 (0.19)	1.33 (1.25)	1.14 (1.01)
	Median (Min, Max)	0.85 (0.5, 1.3)	0.76 (0.5, 1.2)	0.88 (0.1, 7.1)	0.84 (0.1, 7.1)
ALB (g/dL)	Mean (SD)	4.47 (0.25)	3.71 (0.60)	2.63 (0.66)	3.31 (1.03)
	Median (Min, Max)	4.50 (3.8, 5.1)	3.70 (2.6, 4.7)	2.70 (1.2, 4.6)	3.20 (1.2, 5.1)
ALT (U/L)	Mean (SD)	22.05 (11.07)	25.94 (15.15)	42.88 (51.34)	34.84 (41.70)
	Median (Min, Max)	19.00 (6.0, 70.0)	23.50 (5.0, 65.0)	27.00 (3.0, 425.0)	24.00 (3.0, 425.0)
AST (U/L)	Mean (SD)	20.30 (6.35)	38.13 (16.86)	44.23 (49.28)	35.75 (40.19)
	Median (Min, Max)	19.00 (12.0, 47.0)	33.50 (16.0, 69.0)	31.00 (4.0, 437.0)	24.00 (4.0, 437.0)
Race, *n* (%)	White	81 (86.2)	14 (87.5)	117 (70.1)	212 (76.5)
	Black or African American	11 (11.7)	1 (6.3)	15 (9.0)	27 (9.7)
	Asian	1 (1.1)	0 (0.0)	27 (16.2)	28 (10.1)
	American Indian or Alaska Native	1 (1.1)	0 (0.0)	1 (0.6)	2 (0.7)
	Other	0 (0.0)	1 (6.3)	1 (0.6)	2 (0.7)
	Missing	0 (0.0)	0 (0.0)	6 (3.6)	6 (2.2)
Sex, *n* (%)	Male	52 (55.3)	10 (62.5)	106 (63.5)	168 (60.6)
	Female	42 (44.7)	6 (37.5)	61 (36.5)	109 (39.4)

^*a*^ ALB, albumin; ALT, alanine aminotransferase; BSA, body surface area; CrCL, creatinine clearance; IBW, ideal body weight; Max, maximum; Min, minimum; *n*, number of subjects; SCR, serum creatinine; SD, standard deviation.

The patients with candidemia and/or invasive candidiasis who participated in the STRIVE and ReSTORE studies were, in part, critically ill, which probably explains their lower levels of serum albumin. Moreover, these patients were, on average, older than healthy participants. Notably, there were no marked differences in body size measures or the female/male distribution between patients and healthy participants. The majority (26 out of 28) of Asian subjects were enrolled in Study CD101.IV.3.05, which was the only study to include sites in the Asia-Pacific region.

### PK model results

Starting from the previously developed four-compartment PK model (13), structurally simpler two- and three-compartment models with IIV in various parameters were developed and compared. During the initial modeling exercise, eight additional records were identified as outliers using conditional weighted residuals greater than 5 or smaller than −5 as a criterion and were excluded from model development. A three-compartment model with zero-order infusion and first-order elimination was found to be sufficient to describe the data in an unbiased fashion, where its first compartment represents the dosing and observation compartment. The two other compartments were considered peripheral distribution compartments (V2 and V3). When IIVs in V2 and V3 were included in the model, it was recognized that the individual IIVs on V2 and V3 were highly correlated, and, in addition, the typical estimates of V2 and V3 were very similar. Therefore, a model with a shared IIV term and with a single shared parameter for V2 and V3 [peripheral volume (V23)] was tested. Model diagnostics demonstrated accurate individual predictions with this model and did not provide evidence for systematic trends in the weighted residuals. The estimation of a four-compartment model achieved a moderate improvement in the value of the objective function (VOF) (a decrease of 49 points). However, there was no discernible improvement in the goodness-of-fit model diagnostics, and the residual error was nearly identical. Therefore, the three-compartment structural model was considered to adequately describe the PK of rezafungin without overparameterization. The inclusion of IIV terms was tested on various PK parameters, including clearance (CL), central volume of distribution (V1), V23, intercompartmental clearance 1 (Q2), and intercompartmental clearance 2 (Q3). Interindividual variability terms on CL, V1, and V23 were sufficient to achieve good individual predictions. For the residual variability (RV), the constant coefficient of variation (CCV) and combined additive and proportional error models were tested. The simpler CCV model was found to describe the RV adequately.

### Covariate analysis

In the systematic covariate analysis, seven rounds of univariate forward inclusion yielded six covariate effects that were added to the base model: study on CL, infection status on V1, albumin on V23, and body surface area (BSA) on CL, V1, and V23. In the seventh round, none of the tested covariate effects met both criteria of statistical significance (α = 0.01) and a reduction in IIV by 5%. Because of the significant positive correlation of IIVs, the full covariance matrix was included in the model and tested successfully. The VOF improved by 148 points, and the covariance terms were estimated with acceptable precision [18.3% relative standard error expressed as a percent (RSE), 18.5%RSE, and 40.1%RSE]. No covariate was removed from the model during the stepwise-backward elimination process.

During covariate analysis, it was recognized that the estimates for the study effect on CL for Studies CD101.IV.1.01, CD101.IV.1.02, CD101.IV.1.06, and CD101.IV.1.07 were relatively similar, and a model that combined these effects into a single parameter fit the data nearly as well (difference in VOF 4.5 points). In addition, it appeared that the effects estimated for the hepatically impaired subjects from Study CD101.IV.1.15 were similar to those estimated in candidemia and/or invasive candidiasis patients. Moreover, decreased albumin is an indicator of hepatic impairment, and, as expected, it was found to be lower in subjects with hepatic impairment who participated in Study CD101.IV.1.15. Note that decreased albumin is also an indicator of the infection status. Therefore, a new dichotomous category was created that grouped all healthy subjects, including those with normal hepatic function from Study CD101.IV.1.15, together, and all patients with candidemia and/or invasive candidiasis together with subjects with impaired hepatic function from Study CD101.IV.1.15. This latter category was classified as a disease state. Finally, the same categorization was used to replace the covariate effect of infection status on V1 due to the improved interpretability of the model. The goodness-of-fit characteristics of this model were nearly identical to those of the one with infection status on V1 (difference in VOF of 2 points). The final covariates were identified as albumin on V23, BSA on CL, V1, and V23, and disease status on CL and V1.

### Final PK model

**FIG 1:**
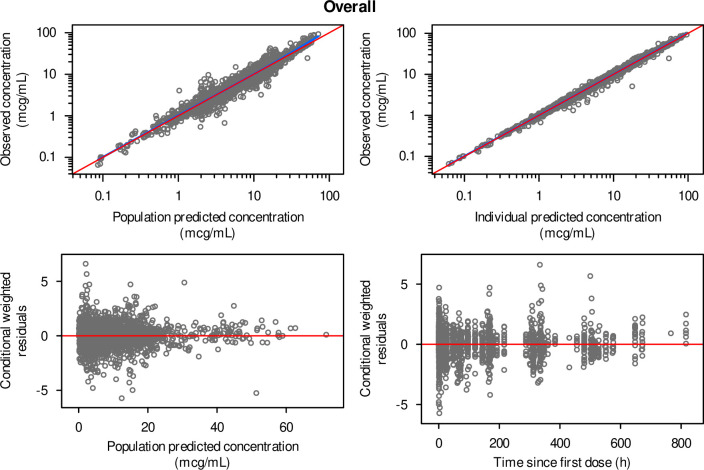
Goodness-of-fit plots of the final population pharmacokinetic model of rezafungin. Note: Red line, reference line; blue curve, smoother line.

The final PK model of rezafungin is a three-compartment model with first-order elimination, characterized by the parameters CL, V1, V23, Q2, and Q3. The variability model included IIV in V1, V23, and CL, their covariances, and a proportional RV model (Table 2). All fixed and random effect parameters, including those of the covariance terms, were estimated with reasonable precision (<39%RSE). The magnitudes of the IIV parameters were moderate for all parameters: 30.5 coefficient of variation expressed as a percent (%CV) for CL, 37.6%CV for V1, and 29.3%CV for V23. Eta shrinkage was low for all parameters (<11%), and the residual error was also low (9.74%CV). Diagnostic plots demonstrate that the model predictions correlate well with the observations and that the conditional weighted residuals do not suggest systematic bias (Fig. 1). In summary, model evaluation demonstrated that the final population PK model of rezafungin adequately described the observed concentration data.

**TABLE 2:** Parameter estimates and standard errors for the final population pharmacokinetic model of rezafungin^*a*^^,*e*,*f*^

Parameter		Final parameter estimate		Magnitude of variability	
		Population mean	%RSE	Final estimate	%RSE
CL	Central clearance (L/h)	0.328	2.77	30.5%CV	13.7
	Proportional shift in CL for healthy	0.276	9.25		
	Exponent of (BSA/1.9) for CL	0.882	16.4		
V1	Central volume (L)	17.7	3.96	37.6%CV	14.7
	Exponent of (BSA/1.9) for V1	1.56	9.47		
	Proportional shift in V1 for healthy	0.222	18.1		
Q2	Distribution clearance 1 (L/h)	0.236	5.38	NE	NA
V23	Peripheral volume 1 and 2 (L)	19.1	2.41	29.3%CV	18.9
	Exponent of (ALB/3.2) for V23	0.708	11.4		
	Exponent of (BSA/1.9) for V23	1.17	14.9		
Q3	Distribution clearance 2 (L/h)	12.4	4.37	NE	NA
cov(IIV in V1, IIV in CL)		0.0560^*b*^	17.3	NA	NA
cov(IIV in V23, IIV in CL)		0.0619^*c*^	18.8	NA	NA
cov(IIV in V23, IIV in V1)		0.0373^*d*^	38.8	NA	NA
Residual variability		0.00949	9.34	9.74%CV	NA
Minimum value of the objective function = 2067.21					

^*a*^ Remark: The covariate effects are defined as follows: $CL=0.328\times {\left(\frac{BSA}{1.9}\right)}^{0.882}\times \left(1+\left(-0.276\times I_{healthy}\right)\right)$, $V1=17.7\times {\left(\frac{BSA}{1.9}\right)}^{1.56}\times \left(1+\left(-0.222\times I_{healthy}\right)\right)$, and $V23=19.1\times {\left(\frac{BSA}{1.9}\right)}^{1.17}\times {\left(\frac{ALB}{3.2}\right)}^{-0.708}$, where $I_{healthy}$ is the indicator variable for healthy subjects who are not infected and have normal liver function (0 = no; 1 = yes).

^*b*^ The calculated correlation coefficient (*r*) associated with cov(IIV in V1, IIV in CL) was 0.516 with *r*^2^ = 0.266.

^*c*^ The calculated correlation coefficient (*r*) associated with cov(IIV in V23, IIV in CL) was 0.723 with *r*^2^ = 0.523.

^*d*^ The calculated correlation coefficient (*r*) associated with cov(IIV in V23, IIV in V1) was 0.357 with *r*^2^ = 0.128.

^*e*^ Abbreviations: ALB, albumin; NA, not applicable; NE, not estimated.

^*f*^ Shrinkage estimates: 3.5% for IIV in CL, 5.2% for IIV in V1, and 10.7% for IIV in V23.

### Clinical relevance

The covariate analysis demonstrated that disease state (defined as candidemia and/or invasive candidiasis patients and hepatically impaired subjects), BSA, and serum albumin are significant predictors of variability in the PK of rezafungin. Covariate levels that lead to lower CL translate into higher exposures. For example, a doubling of BSA from 1.3 to 2.6 m^2^ corresponded to an increase in CL from 0.235 to 0.433 L/h, based on the final model estimates, and healthy subjects had a 27.6% lower CL than infected subjects or those with hepatic impairment (refer to equations in the footer of Table 2).

**FIG 2:**
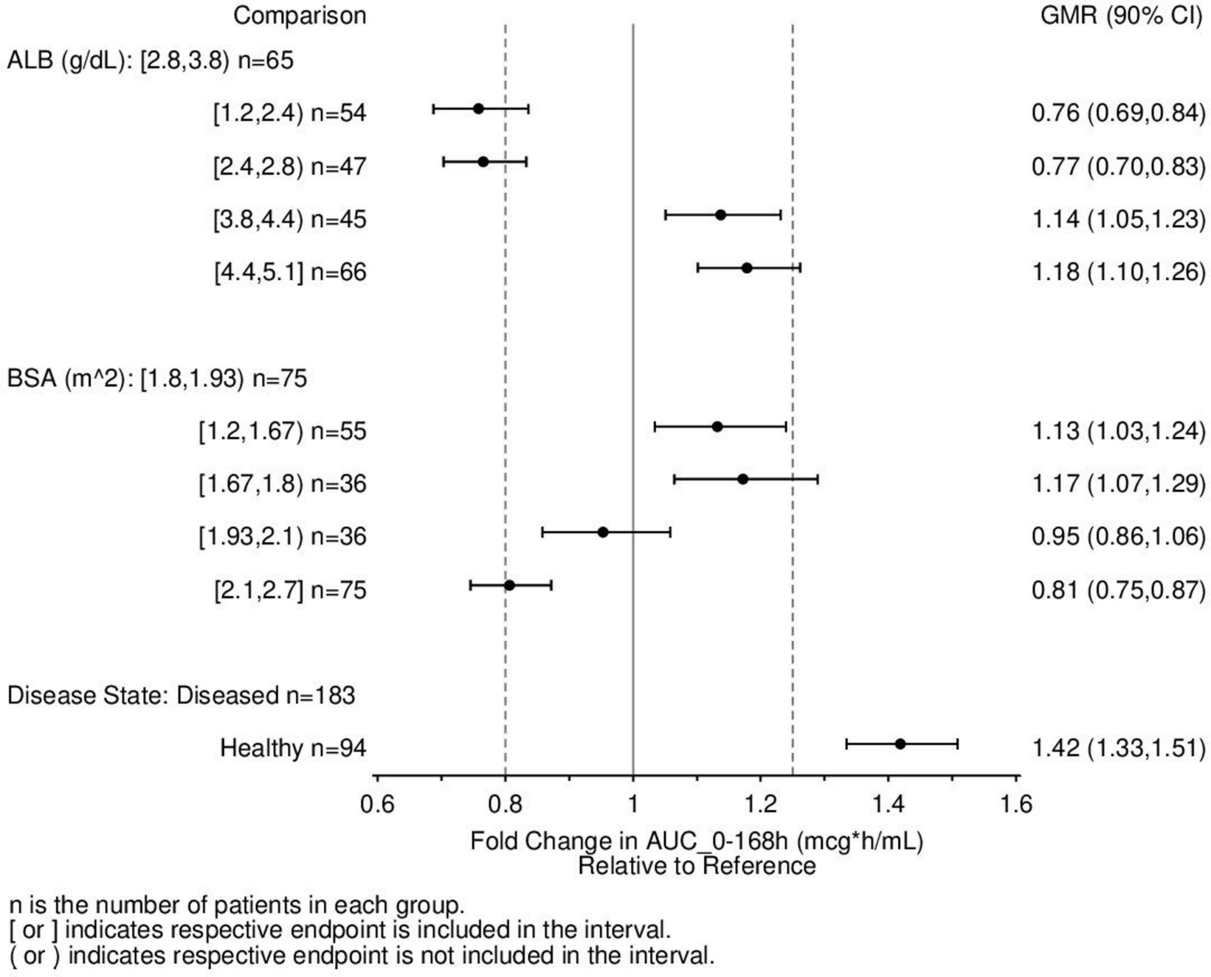
Forest plot illustrating the impact of covariate effects in the final population pharmacokinetic model on rezafungin exposure following a single 400 mg dose for all subjects. ALB, albumin.

To illustrate the relative differences in the impact of the covariates included in the final model on the exposure of rezafungin, forest plots were constructed using simulated exposure for the subjects in the analysis data set, following a single dose of 400 mg. Exposures were summarized by predefined categories or quintiles for each covariate relative to the reference group (selected as the median quintile for albumin and BSA and diseased patients for disease state), with the geometric mean ratios (GMRs) and 90% confidence intervals (CIs) of the exposure measures area under the concentration-time curve from time 0 to 168 h (AUC_0–168h_) and maximum drug concentration (C_max_) depicted in Fig. 2 and Fig. S1, respectively. Though statistically significant and included in the final model, the GMR rezafungin exposure and 90% CI of each covariate effect were not far outside of the default no effect bounds of 0.8 and 1.25.

**FIG 3:**
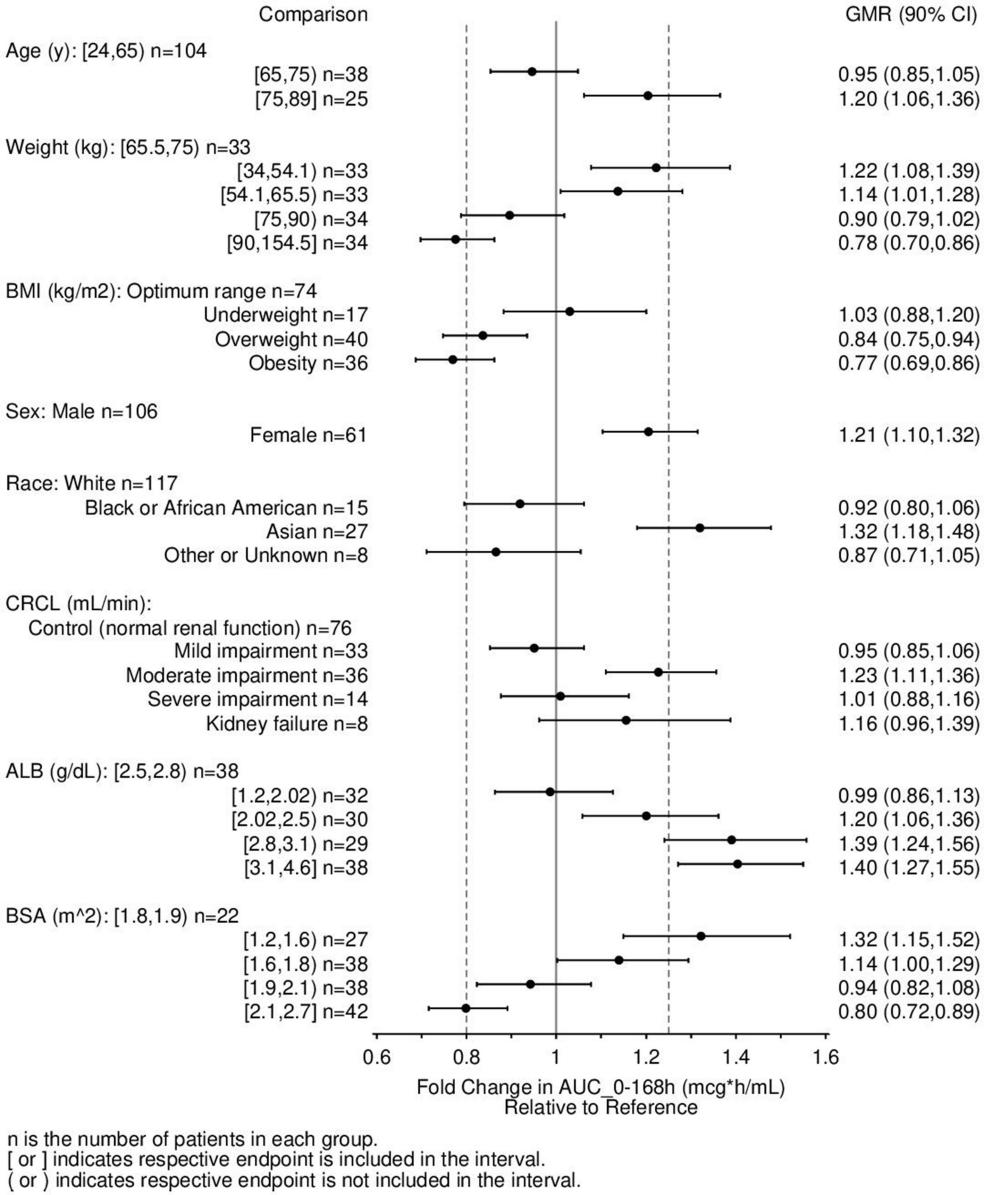
Forest plot illustrating the impact of covariate effects on rezafungin exposure following a single 400 mg dose in patients enrolled in studies CD101.IV.2.03 or CD101.IV.3.05. Definition of BMI groups: optimum range [18.5, 25); underweight <18.5; overweight [25, 30); obese 30 or more. Definition of renal impairment groups based on CrCL: normal renal function ≥90 mL/min, mild impairment 60 to 89 mL/min, moderate impairment 30 to 59 mL/min, severe impairment 15 to 29 mL/min, and kidney failure <15 mL/min. ALB, albumin; CrCL, creatinine clearance.

Additional forest plots were created focusing on the range of exposure predictions of AUC_0–168h_ and C_max_ for the participants of the patient studies (Fig. 3; Fig. S2, respectively) across several patient factors, regardless of whether they were included in the final model or not. Age was not determined to be a significant covariate of rezafungin exposure. Sixty-three patients were aged 65 years or older, with the oldest patient being 89 years old. Thirty-eight patients were aged 65 to <75, and 25 patients were aged 75 to 89 years old. The GMR (90% CI) of AUC_0–168h_ of these two age groups were 0.95 (0.85, 1.05) and 1.20 (1.06, 1.36), respectively, compared to patients 24 to <65 years old (*n* = 104). The change in exposure in patients 65 years or older is not deemed clinically meaningful.

Female subjects appeared to have a 21% higher AUC_0–168h_; however, sex was not identified as a significant independent predictor of PK variability. This was likely because BSA was, on average, lower in female subjects, and BSA was included as a significant covariate in the model.

BSA was identified as a covariate of rezafungin exposure, but it should be noted that other body size measures such as BMI and weight were highly correlated, and the overall fit of the model would be expected to be comparable with any of these as covariates. The impact of a higher BMI on the exposure of rezafungin was investigated due to the clinical utility of this parameter. Stratification of the BMI into underweight (<18.5 kg/m^2^), optimum range (18.5 to <25 kg/m^2^), overweight (25 to <30 kg/m^2^), and obese (≥30 kg/m^2^) identified that the GMR (90% CI) of AUC_0–168h_ of patients classed as overweight (*n* = 40) and obese (*n* = 36) was 0.84 (0.75, 0.94) and 0.77 (0.69, 0.86), respectively, relative to patients with an optimum BMI (*n* = 74). Underweight patients (*n* = 17) had a very similar exposure compared to patients with an optimum BMI [GMR (90% CI) of AUC_0–168h_: 1.03 (0.88, 1.20)]. These changes in exposure are not considered clinically meaningful.

Creatinine clearance (CrCL) was not determined to be a significant covariate of rezafungin exposure. Of the 167 patients in the analysis, 33, 36, and 14 had mild, moderate, and severe renal impairment, respectively. Furthermore, 8 patients had kidney failure, and 76 patients had normal renal function. In the modeling, renal function was defined by CrCL as follows: normal renal function ≥90 mL/min, mild impairment 60 to 89 mL/min, moderate impairment 30 to 59 mL/min, severe impairment 15 to 29 mL/min, and kidney failure <15 mL/min. Comparison of the AUC_0–168h_ in patients with renal impairment (including kidney failure) with patients with normal renal function identified that the GMR (90% CI) range was 0.95 (0.85, 1.06), 1.23 (1.11, 1.36), 1.01 (0.88, 1.16), and 1.16 (0.96, 1.39) for patients with mild, moderate, and severe renal impairment and kidney failure, respectively, range was 0.95 (Fig. 3). This change in exposure in patients with renal impairment is not clinically meaningful.

In summary, the GMRs and 90% CIs of the exposure measures for all covariates were well within the 0.5 to 2.0 range, which suggests that no dose adjustment is necessary, even for patients with exceptional covariate levels. Summary statistics of exposure measures after the first 400 mg dose of rezafungin, stratified by health status, are provided in Table 3.

**TABLE 3:** Summary statistics of exposure measures for 400 mg of a single dose of rezafungin, stratified by health status^*a*^^,^^*b*^

Parameter	Statistic	Healthy subjects, *n* = 94	Hepatic impairment, uninfected, *n* = 16	Patients, *n* = 167
AUC_0–168h_ (μg × h/mL)	Mean (SD)	1,091.512 (215.164)	791.452 (152.248)	798.607 (295.120)
	Geometric mean (geometric %CV)	1,070.513 (20.149)	778.250 (19.012)	752.507 (35.100)
C_max_ (μg/mL)	Mean (SD)	22.244 (4.789)	17.291 (3.017)	18.761 (6.448)
	Geometric mean (geometric %CV)	21.725 (22.466)	17.061 (16.797)	17.715 (35.418)
C_min, 168h_ (μg/mL)	Mean (SD)	3.329 (0.741)	2.176 (0.533)	2.292 (1.072)
	Geometric mean (geometric %CV)	3.247 (22.858)	2.113 (25.916)	2.097 (43.351)
Clearance (L/h)	Mean (SD)	0.234 (0.048)	0.343 (0.077)	0.350 (0.130)
	Geometric mean (geometric %CV)	0.229 (20.266)	0.336 (21.829)	0.328 (38.396)
Total volume of distribution at steady state (L)	Mean (SD)	43.410 (10.265)	57.264 (10.702)	67.451 (27.744)
	Geometric mean (geometric %CV)	42.312 (22.744)	56.279 (19.744)	62.434 (40.964)
Half-life (h)	Mean (SD)	144.039 (14.027)	133.372 (12.249)	151.960 (29.086)
	Geometric mean (geometric %CV)	143.368 (9.737)	132.827 (9.457)	149.458 (18.095)

^*a*^ C_max_, maximum observed drug concentration; C_min, 168h_, minimum observed drug concentration from time 0 to 168 h; n, number of subjects; SD, standard deviation.

^*b*^ Individual clearance parameters were model-based estimates (CL). The individual total volume of distribution at steady state was calculated as the sum of the individual model-based estimates of the central and the peripheral volumes of distribution. The individual half-life (*T*_1/2_) was estimated from simulated concentrations during week 2 after the last dose using the formula *T*_1/2_ *=* log(2) / *sl*, where log is the natural logarithm and *sl* is the slope of the regression line of the log-transformed simulated concentrations versus time.

### Target attainment simulations

A total of 100,000 virtual patients with *Candida* infections were randomly resampled from patients enrolled in Studies CD101-IV-2-03 (STRIVE) and CD101-IV-3-05 (ReSTORE). The distributions of significant PK covariates (that is, baseline albumin and baseline BSA) were similar between observed and virtual patients. Day 1 *f*AUC_0–168h_ values for 100,000 virtual patients administered 400 mg rezafungin followed by 200 mg weekly for 3 weeks were simulated.

**FIG 4:**
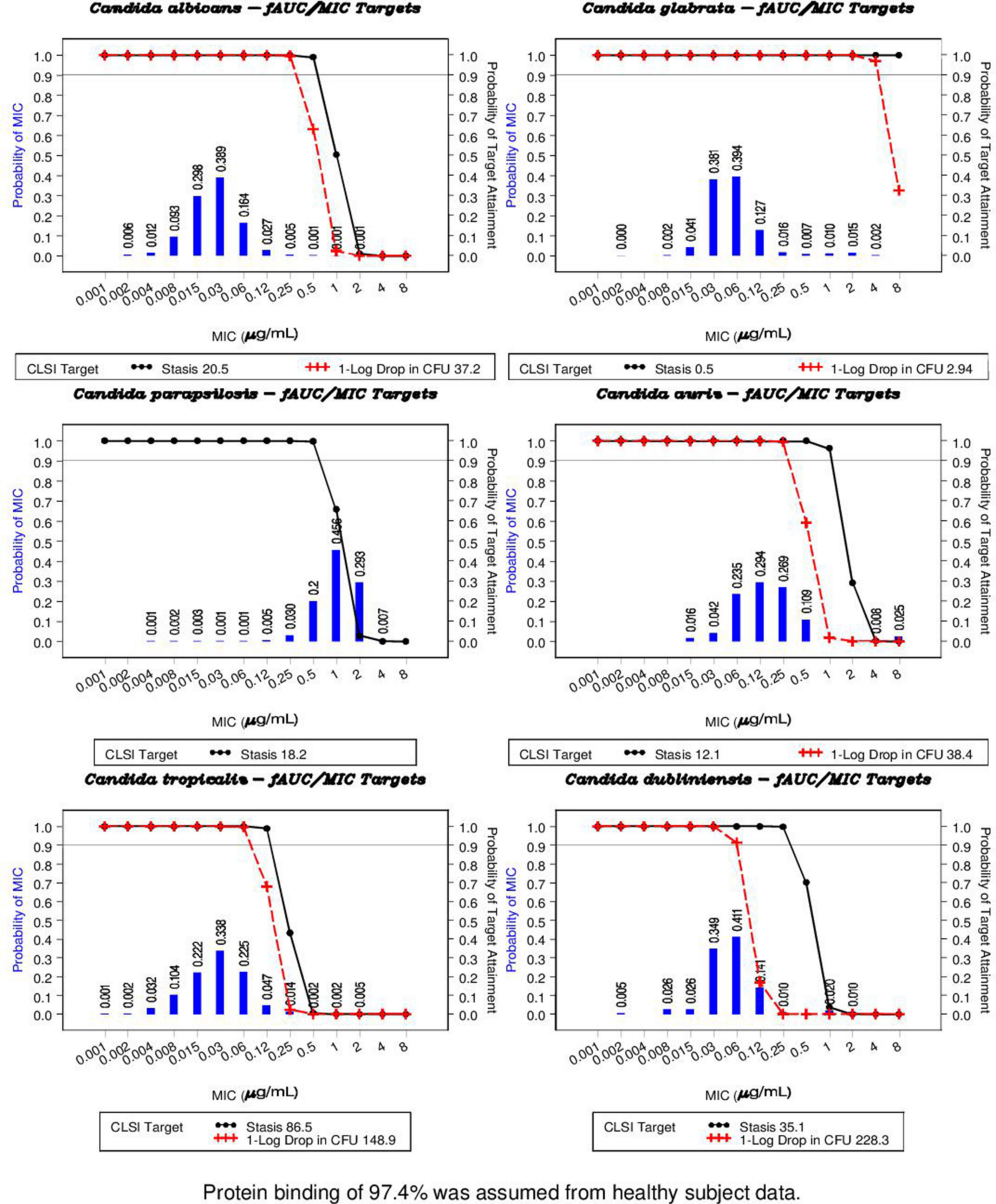
Probability of pharmacokinetic/pharmacodynamic target attainment for 400 mg rezafungin against *Candida* species using CLSI methodology. *f*, free.

**FIG 5:**
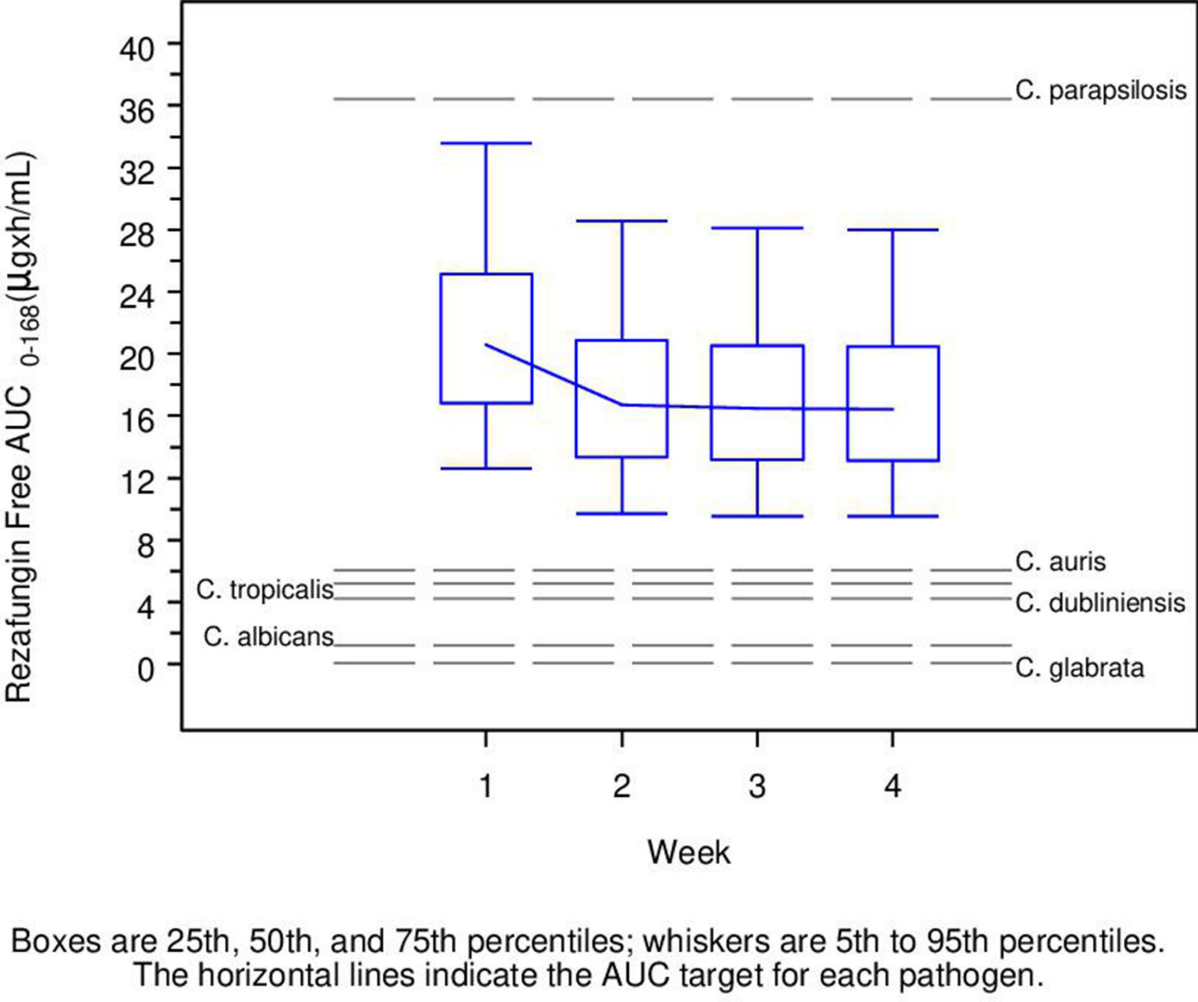
Boxplots of rezafungin *f*AUC_0–168h_ versus week in patients administered 400 mg for 1 week, followed by 200 mg for 3 weeks. Dotted lines indicate the *f*AUC_0–168h_ required to achieve stasis at MIC_90_ for each *Candida* species.

Figure 4 shows the estimated PK/PD target attainment for stasis and 1-log drop in CFU using *f*AUC_0–168h_ values following the 400 mg loading dose and CLSI methodology to calculate MIC over the *Candida* species MIC distribution (19–28), separately for each species. The bars represent the observed distribution of MIC values in the surveillance data. The gray horizontal line represents the simulated probability of target attainment. For *C. albicans*, the simulated probabilities of target attainment for both targets were >90% at MIC values ≤0.25 µg/mL, and the probability approached zero at an MIC value of 1 µg/mL or greater for a 1-log drop in CFU and above 1 µg/mL for stasis. For *C. glabrata*, the simulated probabilities of target attainment for both targets were >90% at MIC values ≤4 µg/mL. For *C. parapsilosis*, the simulated probabilities of target attainment for the stasis target were >90% at MIC values ≤0.5 µg/mL. For *C. auris*, the simulated probabilities of target attainment for both targets were >90% at MIC values ≤0.25 µg/mL. For *C. tropicalis*, the simulated probabilities of target attainment for both targets were >90% at MIC values ≤0.06 µg/mL. For *C. dubliniensis*, the simulated probabilities of target attainment for both targets were >90% at MIC values ≤0.06 µg/mL. Figure 5 provides the boxplots of *f*AUC_0–168h_ versus week for patients administered 400 mg for 1 week, followed by 200 mg for 3 weeks. The horizontal lines represent the *f*AUC_0–168h_ target for stasis at the MIC_90_ for the various *Candida* species (19–28).

## DISCUSSION

Based on the pooled data from seven clinical studies in healthy subjects, hepatically impaired subjects, and patients with candidemia and/or invasive candidiasis, a population PK model for rezafungin was developed. The model found to best describe the available data was a three-compartment model with first-order elimination characterized by the parameters CL, V1, V23, Q2, and Q3, with random variability in the three parameters CL, V1, and V23, and a proportional error model. Model evaluation demonstrated that this model, which was similar but structurally simpler than the previously developed four-compartment model, adequately described the data with similar exposure estimates (2).

The impact of intrinsic and extrinsic factors on the PK variability of rezafungin was evaluated using an extensive list of potential covariate effects. The systematic search revealed the following statistically significant predictors of PK variability: albumin on V23; BSA on CL, V1, and V23; and disease status (defined as candidemia and/or invasive candidiasis patients and hepatically impaired subjects) on CL and V1. The finding that both albumin levels and disease state were significant covariates in the population PK model is likely to be related, as, in general, albumin levels were reduced in subjects with hepatic impairment as well as in subjects infected with candidemia and/or invasive candidiasis. However, for subjects in the diseased category and subjects with low albumin levels, the reduction in exposure was not considered to be clinically meaningful, and no dose adjustments were required in these subjects. None of the other tested factors, including age, sex, race, or CrCL, were found to be statistically significant predictors of rezafungin PK.

Although BSA was identified as a significant covariate in the population PK model, when considering patients that were underweight, overweight, or obese, as categorized by BMI, the exposure change in these groups of patients was not considered clinically meaningful and, therefore, there were no dose adjustments required in patients based on BMI.

Approximately 40% (63/167) of the patients included in the modeling were aged 65 years and older, with the oldest patient being 89 years old. A total of 38 subjects were included in the 65- to 74-year-old age group, 18 subjects were included in the 75- to 84-year-old age group, and 7 subjects were included in the >85-year-old age group. This enabled a robust exploration of the impact of age on the exposure of rezafungin in patients, which showed that there was no requirement to dose adjust in elderly patients. Similarly, 54% of patients (91/167) had renal impairment ranging from mild impairment to kidney failure, enabling assessment of the impact of renal impairment on exposure to rezafungin. This analysis showed that the change in exposure in patients with renal impairment was not clinically meaningful, and no dose adjustments were required in these subjects.

Rezafungin had a high probability of target attainment, covering the majority of the observed MIC distributions for most organisms tested, which represented the vast majority of clinically relevant pathogens and emerging threats. The probability of attaining the PK/PD target (*f*AUC_0–168h_/MIC ratio) for stasis and a 1-log drop in CFU following a 400-mg dose of rezafungin IV was achieved against five of the six *Candida* species tested. For example, the simulated probabilities of target attainment for both stasis and 1-log kill were >90% at MIC values ≤0.25 µg/mL for *C. albicans*, and >90% at MIC values ≤4 µg/mL for *C. glabrata*. These results demonstrated rezafungin coverage for the majority of the MIC distributions in most cases at multiple dilutions above the MIC_90_ values for those organisms and are consistent with previously reported results (18).

For *C. parapsilosis*, rezafungin only achieved stasis in nonclinical models, and this target was not achieved at a high probability within the usual range of MICs. This has been observed for other echinocandins and appears to be limited to nonclinical testing as *C. parapsilosis* responds well clinically to echinocandin therapy despite the intrinsically elevated MICs of this species (29). It is believed that the *C. parapsilosis* strains utilized in the model (more than 80% of which were not suitable) are highly fit or virulent and therefore may result in elevated targets (30).

Of the 92 rezafungin-treated Phase 2 and 3 patients included in the PK/PD analysis, 96% of patients (*N* = 88) achieved their *f*AUC_0–168h_/MIC target ratios, including 100% of the non-parapsilosis *Candida* spp. There were a total of eight cases of *C. parapsilosis* in the data set, four of these patients did not hit their target with MIC values of 2 (*N* = 3) and 1 (*N* = 1). However, all four were cured on both days 5 and 14. Of the four patients who did meet the *f*AUC_0–168h_/MIC target, two responded on both days 5 and 14, and two did not respond on days 5 or 14. Given these results, there was no observed relationship between *f*AUC_0–168h_/MIC and *C. parapsilosis*.

It is noted that the target attainment (TA) methodology uses the simulated *f*AUC_0–168h_/MIC ratios; therefore, the value of plasma protein binding (PPB) influences this analysis. The free fraction of rezafungin used in the analysis was 2.6%, obtained from measuring PPB in plasma from healthy subjects. This value was selected as a conservative figure as the measured PPB in infected patients was variable and approximately three times higher in infected patients. While there are still questions in the scientific community about how to verify information from the nonclinical model to support PK/PD-based translation of an antifungal drug’s effectiveness from animal infection models to humans, it is acknowledged that the TA analysis can provide valuable insight into dose selection for clinical studies and, as such, was successfully used to support the selection of the dose used in the Phase 2 and Phase 3 clinical studies for rezafungin.

In summary, a population PK model has been successfully developed for rezafungin that adequately describes the observed concentration data. The identified covariates BSA, disease state, and albumin and their impact on the PK of rezafungin were very similar to what was reported previously in smaller data sets (13, 14), with no clinically meaningful changes in PK expected across a wide range of patient factors including sex, BMI, renal function, and age. Therefore, no dose adjustments are required based on these covariates. The population PK model was successfully used to simulate exposures in a virtual population of patients that were used to assess the probability of target attainment against six *Candida* species. Rezafungin target attainment results indicated that therapeutic drug exposures should be achieved against relevant *Candida* species for most subjects.

In conclusion, based on the population PK analysis, a common dose regimen of rezafungin is adequate for all adult patients.

## MATERIALS AND METHODS

### Data

Data were obtained from five Phase 1 studies (CD101.IV.1.01, CD101.IV.1.02, CD101.IV.1.06, CD101.IV.1.07, and CD101.IV.1.15), a Phase 2 study (CD101.IV.2.03; STRIVE), and a Phase 3 study (CD101.IV.3.05; ReSTORE) (Table S1). All study participants met the inclusion and exclusion criteria for the study in which they were enrolled. All clinical studies were conducted in compliance with International Council for Harmonisation guidelines on Good Clinical Practice.

### Study design

Dosing regimens for rezafungin are described in Table S1. Data from both single- and multiple-dose studies, with doses ranging from 50 to 1,400 mg, were included in this analysis. All doses of rezafungin were administered via IV infusion.

Efficacy and safety of rezafungin for the treatment of patients with candidemia and/or invasive candidiasis were evaluated in a Phase 2 multicenter prospective, randomized, double-blind comparator study of rezafungin (IV; Group 1: 400 mg loading dose the first week followed by 400 mg once weekly for a total of two to four doses, or Group 2: 400 mg loading dose the first week followed by 200 mg once weekly for a total of two to four doses) versus IV caspofungin with the option for oral fluconazole step down therapy (STRIVE trial; NCT02734862).

The Phase 3 efficacy study (ReSTORE; NCT03667690) was a multicenter, prospective, randomized, double-blind, double-dummy, efficacy, and safety study of rezafungin (IV; 400 mg loading dose the first week followed by 200 mg once weekly for a total of two to four doses) versus caspofungin (IV) followed by optional oral fluconazole step-down therapy (in qualifying subjects) in subjects with candidemia and/or invasive candidiasis.

Dense PK samples were collected in the Phase 1 studies, and a mixture of dense and sparse PK samples were collected in the Phase 2 and Phase 3 studies.

Rezafungin for injection is a lyophilized powder of rezafungin acetate. The molecular weight is 1,226.39 g/mol for the free peptide and 1,285.44 g/mol for the acetate salt. No adjustment for salt form was necessary. The dose of rezafungin was expressed in milligram (mg) units.

Rezafungin plasma concentrations were assessed in different laboratories using bioanalytical liquid chromatography with tandem mass spectrometry methods. Rezafungin concentrations were reported and analyzed in microgram per milliliter (μg/mL) units.

### Population pharmacokinetic analysis methodology

The overall procedures for the development of a population PK model for rezafungin included exploratory data analysis, base structural model development, evaluation of covariate effects, final model refinement, and model qualification. Using the software NONMEM version 7.3, the first-order conditional estimation with interaction method was applied during all stages of the model development process (31).

The variability model was developed, including and testing IIV in PK parameters using exponential random effect models assuming a log normal distribution of each of the parameters. The residual variability in the observed concentrations was described using a proportional (constant coefficient of variation) error model or, alternatively, using a combination additive plus proportional error model.

A systematic stepwise covariate analysis was conducted to assess the ability of intrinsic or extrinsic factors to explain portions of the IIV for select PK parameters. A stepwise univariate forward selection (significance criterion: change in VOF α = 0.01, 1 df, and reduction of IIV of at least 5%), followed by stepwise backward elimination (significance criterion: change in VOF α = 0.001, 1 df) technique, was utilized. The following stationary covariates were evaluated: age, body weight, BMI, BSA, sex, race, serum creatinine, creatinine clearance (32), albumin, liver parameters alanine transaminase and AST, infection status, assay, Acute Physiology and Chronic Health Evaluation disease score, study, and dose. Covariate analysis was performed using a systematic stepwise forward selection and backward elimination approach. In addition, the variance-covariance structure of IIV and the distribution of the residuals were investigated to identify an appropriate variability model.

The final and intermediate models were evaluated using the following criteria: the VOF, which is a statistic that is proportional to minus twice the log likelihood of the data; the precision of the parameter estimates; the overall reduction in IIV and RV; the robustness of the fit of the model to the data both visually and statistically; standard numerical diagnostics; and graphical goodness-of-fit characteristics.

The final PK model of rezafungin, the individual empirical Bayesian PK parameter estimates, and the individual dosing histories were used to simulate dense PK profiles for each subject using the R package mrgsolve (33). Numerical integration was performed to calculate PK exposure measures: AUC_0–168h_, or time of next dose if the next dose is given before 168 h; C_max_; and minimum observed drug concentration from time 0 to 168 h (C_min, 168h_) for each subject.

### Target attainment simulation methodology

The goals of the target attainment analyses were to estimate the probability of achieving PK/PD targets across the range of MIC values for six *Candida* species based on CLSI methodology, as shown in Table 4. Using the final rezafungin population PK model and the distributions of the demographic covariates (determined to be significant predictors of rezafungin PK) of candidemia and/or invasive candidiasis patients enrolled in Studies CD101-IV-2-03 (STRIVE trial; NCT02734862) and CD101-IV-3-05 (ReSTORE trial; NCT03667690), a total of 100 trials of 1,000 virtual patients each (100,000 patients total) were simulated. Vectors of covariates were randomly resampled from the observed Phase 2 and Phase 3 distributions and assigned to the 100,000 virtual patients. The virtual patients were assigned the dosing regimen of interest (IV rezafungin: 400 mg for week 1, followed by 200 mg weekly for 3 weeks). All fixed and random effect parameters were fixed to the final estimates, and individual Bayesian estimates of PK parameters were simulated for each patient.

**TABLE 4:** Nonclinical pharmacokinetic/pharmacodynamic targets, by species^*a*^^,^^*b*^^,^^*c*^

Method	Genus	Species	Stasis	1-Log drop	Source
			*f*AUC_0–168h_/MIC median	*f*AUC_0–168h_/MIC median	
CLSI	*Candida*	*albicans*	20.5	37.2	1
		*glabrata*	0.5	2.94	1
		*parapsilosis*	18.2	NA	1
		*auris*	12.1	38.4	2
		*tropicalis*	86.5	148.9	3
		*dubliniensis*	35.1	228.3	3

^*a*^ NA, not applicable.

^*b*^ The maximum effect was stasis for *Candida parapsilosis*. For *Candida auris* values, the published *f*AUC_0–24 h_/MIC target was multiplied by 7 to get a weekly ratio to match all the rest.

^*c*^ Source: ^1^Lepak AJ, Zhao M, VanScoy B, Ambrose PG, Andes DR. Pharmacodynamics of a long-acting echinocandin, CD101, in a neutropenic invasive-candidiasis murine model using an extended-interval dosing design. Antimicrob Agents Chemother. 2018 Jan 25;62(2):e02154-17; ^2^Lepak AJ, Zhao M, Andes DR. Pharmacodynamic evaluation of rezafungin (CD101) against *Candida auris* in the neutropenic mouse invasive candidiasis model. Antimicrob Agents Chemother. 2018 Oct 24;62(11):e01572-18; ^3^Lepak AJ, Zhao M, Andes DR. Determination of pharmacodynamic target exposures for rezafungin against *Candida tropicalis* and *Candida dubliniensis* in the neutropenic mouse disseminated candidiasis model. Antimicrob Agents Chemother. 2019 Oct 22;63(11):e01556-19.

Using each simulated patient’s Bayesian PK parameters and dose amounts, simulations were performed to obtain plasma concentration profiles. R software was used to integrate the predicted concentration-time profiles to obtain weekly estimates of AUC_0–168h_. For the purposes of target attainment analyses, AUC_0–168h_ after the first dose was used (day 1). Protein binding of 99.2% in mouse plasma and of 97.4% in human (presumed healthy) plasma has been reported (34). Therefore, 2.6% was used to adjust for unbound free drug AUC_0–168h_ and to calculate separate *f*AUC_0–168h_/MIC ratio variables for the range of MIC values from *Candida* species collected in patients with candidemia and/or invasive candidiasis enrolled in Studies CD101-IV-2-03 (STRIVE trial; NCT02734862) and CD101-IV-3-05 (ReSTORE trial; NCT03667690).

Percent probabilities of achieving the nonclinical PK/PD targets associated with net fungal stasis and a 1-log10 drop in CFU reductions from baseline (if available) for *C. albicans*, *C. glabrata*, *C. parapsilosis*, *C. auris*, *C. tropicalis*, and *C. dubliniensis* were calculated for the rezafungin regimen (400 mg, day 1) using microbiology results based on CLSI methodologies. The mean target attainment for net fungal stasis and 1-log10 CFU reductions was compared to the frequency distribution of MIC values from surveillance results by species. The PK/PD target attainment graphs for each species were generated by plotting the mean probability of simulated *f*AUC_0–168h_/MIC ratios above each PK/PD target, across all 100 data sets, for net fungal stasis and 1-log10 CFU reductions. The MIC susceptibility breakpoint was determined to be the highest clinically relevant MIC value with a probability of PK/PD target attainment of at least 0.9.

## SUPPLEMENTAL MATERIAL

Supplemental file 1 aac.00916-23-s0001.docx Fig. S1 and S2 and Table S1.

ASM does not own the copyrights to Supplemental Material that may be linked to, or accessed through, an article. The authors have granted ASM a non-exclusive, world-wide license to publish the Supplemental Material files. Please contact the corresponding author directly for reuse.
