# Supplementary material for: Population pharmacokinetic modeling and target attainment analyses of rezafungin for the treatment of candidemia and invasive candidiasis
Source: Antimicrob Agents Chemother. 2023 Nov 28;67(12):e00916-23. doi: 10.1128/aac.00916-23 (PMC10720538; doi:10.1128/aac.00916-23)
Supplement: Supplemental file 1 — Fig. S1 and S2 and Table S1. [file aac.00916-23-s0001.docx]

SUPPLEMENTAl Material

Forest plots of Cmax illustrating the effect of covariates are provided below for all subjects and patients.

Figure S-1. Forest plots illustrating the impact of covariate effects in the final population pharmacokinetic model on rezafungin C_max_ following a single 400-mg dose, all subjects


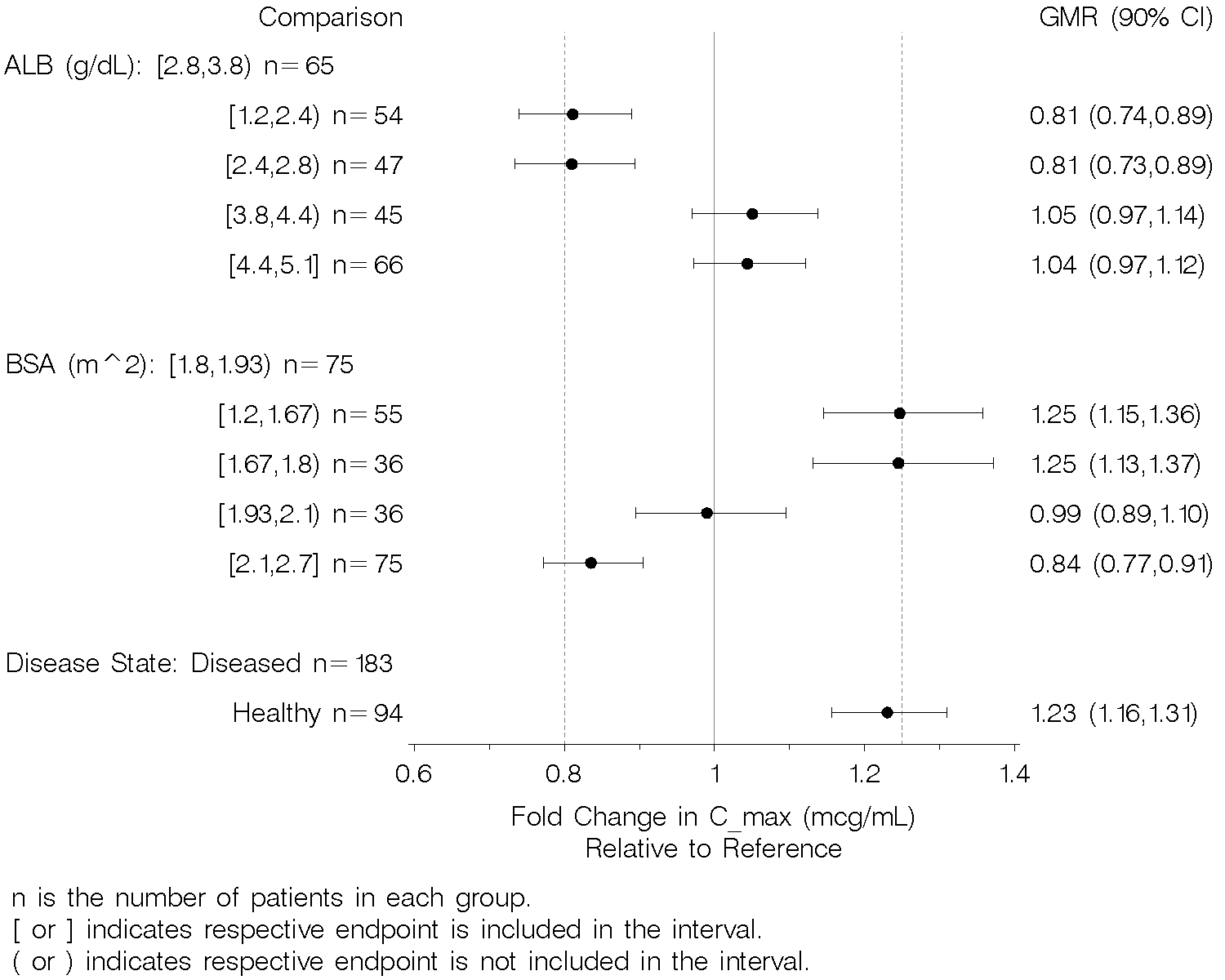


Abbreviations: ALB, albumin; BSA, body surface area; CI, confidence interval; Cmax, maximum drug concentration; GMR, geometric mean ratio.

Figure S-2. Forest plots illustrating the impact of covariate effects on rezafungin C_max_ following a single 400-mg dose in patients enrolled in studies CD101.IV.2.03 or CD101.IV.3.05


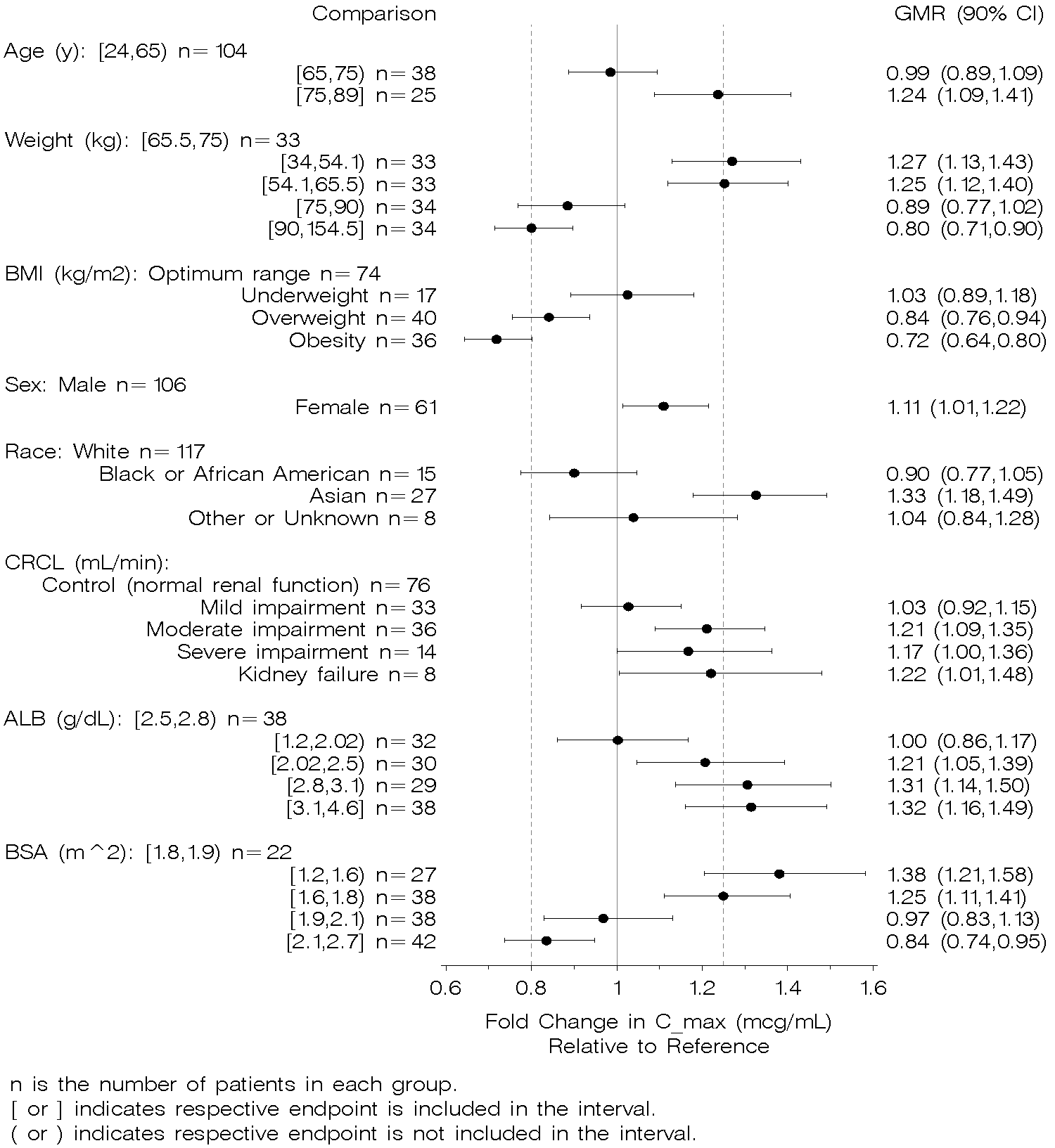


Definition of BMI groups: optimum range [18.5, 25); underweight < 18.5; overweight [25, 30); obese 30 or more. Definition of renal impairment groups based on CrCL: normal renal function ≥ 90 mL/min, mild impairment 60 to 89 mL/min, moderate impairment 30 to 59 mL/min, severe impairment 15 to 29 mL/min, and kidney failure < 15 mL/min.

Abbreviations: ALB, albumin; BSA, body surface area; CI, confidence interval; Cmax, maximum drug concentration; CrCL, creatinine clearance; GMR, geometric mean ratio.

| Table S-1. Clinical studies included in the analysis | | | |
| --- | --- | --- | --- |
| Study Number Phase | Study Title | Participants | Dosing Regimen |
| CD101.IV.1.01 Phase 1 | A Phase 1, randomized, double-blind, single-dose, dose escalation study to determine the safety, tolerability, and pharmacokinetics of CD101 injection in healthy subjects | 32 subjects; 4 cohorts of 8 subjects (6 active, 2 placebo) | Single doses of 50, 100, 200, or 400 mg rezafungin or placebo infused IV over 1 hour |
| CD101.IV.1.02 Phase 1 | A Phase 1, randomized, double-blind, multiple-dose, dose-escalation study to determine the safety, tolerability, and pharmacokinetics of CD101 injection in healthy subjects | 24 subjects; 3 cohorts of 8 subjects (6 active, 2 placebo) | Cohorts 1 and 2: 2 weekly doses of 100 or 200 mg rezafungin infused IV over 1 hour  Cohort 3: 3 weekly doses of 400 mg rezafungin infused IV over 1 hour |
| CD101.IV.1.06 Phase 1 | A Phase 1, randomized, double-blind, comparative, placebo and positive controlled study to evaluate the safety, pharmacokinetics, and effects on the electrocardiogram of CD101 for injection in healthy subjects | 60 subjects; 2 cohorts of 30 subjects | Single dose of 600 or 1400 mg rezafungin or placebo. 600-mg dose infused over 1.5 hours followed by IV placebo infusion over 2 hours; 1400-mg dose infused as a divided dose of 375 mL over 1.5 hours followed by a 500-mL infusion over 2 hours: oral dosing of moxifloxacin (400 mg) as positive control |
| CD101.IV.1.07 Phase 1 | A Phase 1, multiple-dose, assessor-blinded study to determine the photosensitivity of CD101 for injection in healthy subjects | 24 subjects (12 CD101, 12 placebo) | 4 weekly doses of 400 mg rezafungin or placebo infused IV over 1 hour |
| CD101.IV.1.15 Phase 1 | An open-label, single-dose, Phase 1 study to evaluate the safety, tolerability, and pharmacokinetics of rezafungin in adult subjects with hepatic impairment relative to matched controls | Stage 1 – 16 subjects: 8 subjects with moderate hepatic impairment (Group 1a); 8 healthy adults with normal hepatic function (Group 1b) matched to hepatic impaired subjects in Group 1a  Stage 2 – 16 subjects: 8 subjects with severe hepatic impairment (Group 2a); 8 healthy adults with normal hepatic function (Group 2b) matched to hepatic impaired subjects in Group 2a | IV infusion of 400 mg rezafungin over 1 hour |
| CD101.IV.2.03; STRIVE trial; NCT02734862 Phase 2 | A Phase 2, multicenter, randomized, double-blind study of the safety, tolerability, and efficacy of intravenous CD101 vs intravenous caspofungin followed by oral fluconazole step-down in the treatment of subjects with candidemia and/or invasive candidiasis | Part A: 107 subjects  Part B: 100 subjects | Part A:  Group 1: 400 mg rezafungin Week 1, 400 mg rezafungin Week 2, followed by 2 optional weekly doses of 400 mg rezafungin administered IV over 1 hour  Group 2: 400 mg of rezafungin Week 1 followed by 200 mg rezafungin Week 2, followed by up to 2 optional weekly doses of 200 mg rezafungin administered IV over 1 hour  Part B:  Group 2: 400 mg rezafungin Week 1 followed by 200 mg rezafungin Week 2, followed by up to 2 optional weekly doses of 200 mg rezafungin administered IV over 1 hour  In both Part A and Part B, subjects may receive oral step-down therapy of placebo after ≥ 3 days of IV therapy |
| CD101.IV.3.05; ReSTORE trial; NCT03667690 Phase 3 | A Phase 3, multicenter, randomized, double-blind study of the efficacy and safety of rezafungin for injection versus intravenous caspofungin followed by optional oral fluconazole step-down in the treatment of subjects with candidemia and/or invasive candidiasis (the ReSTORE study) | 218 subjects (109 rezafungin, 109 caspofungin) | 400 mg of rezafungin Week 1, 200 mg rezafungin Week 2, followed by up to 2 optional weekly doses of 200 mg rezafungin administered IV over 1 hour. Subjects may receive oral step-down therapy of placebo after ≥ 3 days of IV therapy |
